# Correction: BatCount: A software program to count moving animals

**DOI:** 10.1371/journal.pone.0305183

**Published:** 2024-06-05

**Authors:** Ian Bentley, Vona Kuczynska, Valerie M. Eddington, Mike Armstrong, Laura N. Kloepper

In the Materials and methods section, the URL for accessing the software install file, source code, and user guide is incorrect. The correct URL is: https://sourceforge.net/projects/batcount/.
